# Multicystic meningioangiomatosis

**DOI:** 10.1186/1471-2377-14-32

**Published:** 2014-02-20

**Authors:** Peifeng Li, Guangbin Cui, Yingmei Wang, Ming Geng, Zhe Wang

**Affiliations:** 1 Department of Pathology, General Hospital of Jinan Military Command, Ji’nan 250031, China; 2 Department of Pathology, State Key Laboratory of Cancer Biology, Xijing Hospital and School of Basic Medicine, Fourth Military Medical University, Xi’an 710032, China; 3 Department of Radiology, Tangdu Hospital, Fourth Military Medical University, Xi’an 710038, China

**Keywords:** Meningioangiomatosis, Multicystic, Seizure, Hamartomatous lesion

## Abstract

**Background:**

Meningioangiomatosis (MA) is a rare hamartomatous lesion. Only six cases of cystic MA have been reported in the literature.

**Case presentation:**

We present a case of multicystic MA. A 21-year-old woman without any stigmata of neurofibromatosis type 2 presented with intractable seizures since 10 years. Brain magnetic resonance imaging revealed a well-defined, multicystic mass with heterogeneous signal intensity in the right temporal lobe. The patient underwent resection of the lesion and of the epileptogenic cortex under intraoperative electrocorticography (ECoG) assistance. Histopathological examination showed proliferation of perivascular cells that were arranged in a cuff pattern and were positive for vimentin, D2-40 and smooth muscle actin. Mutiple microcysts and enlarged perivascular spaces were present, which was similar to the structure of the arachnoid cavity. Hyalinized collagen fibers with round concentric acellular eosinophilic lamellae within areas of reactive gliosis were noted for the first time in MA. The patient was followed up without any clinical symptoms or recurrence for 2 years.

**Conclusion:**

MA may originate from arachnoid and vascular tissue trapped in the cortical parenchyma during brain development, and the cysts may have resulted from the gradual accumulation of cerebrospinal fluid in the perivascular spaces of the trapped tissue. Resection of the lesion and of the epileptogenic cortex is important not only for pathological diagnosis but also for seizure control, and intraoperative ECoG assistance is recommended.

## Background

Meningioangiomatosis (MA) is a rare and benign congenital disorder that involves the cortex and the overlying leptomeninges and primarily affects children and young adults [1]. It may occur sporadically or in association with neurofibromatosis type 2 (NF2) [2]. Approximately 120 cases of MA have been reported in the literature. Of these, only six cases were accompanied by cystic lesions [3-7]. We assess the clinical, eletrophysiological, imaging and pathological features of a case of sporadic MA with multiple microcysts, and herein, we discuss the mechanisms and treatment of this disorder.

## Case presentation

**Figure 1:**
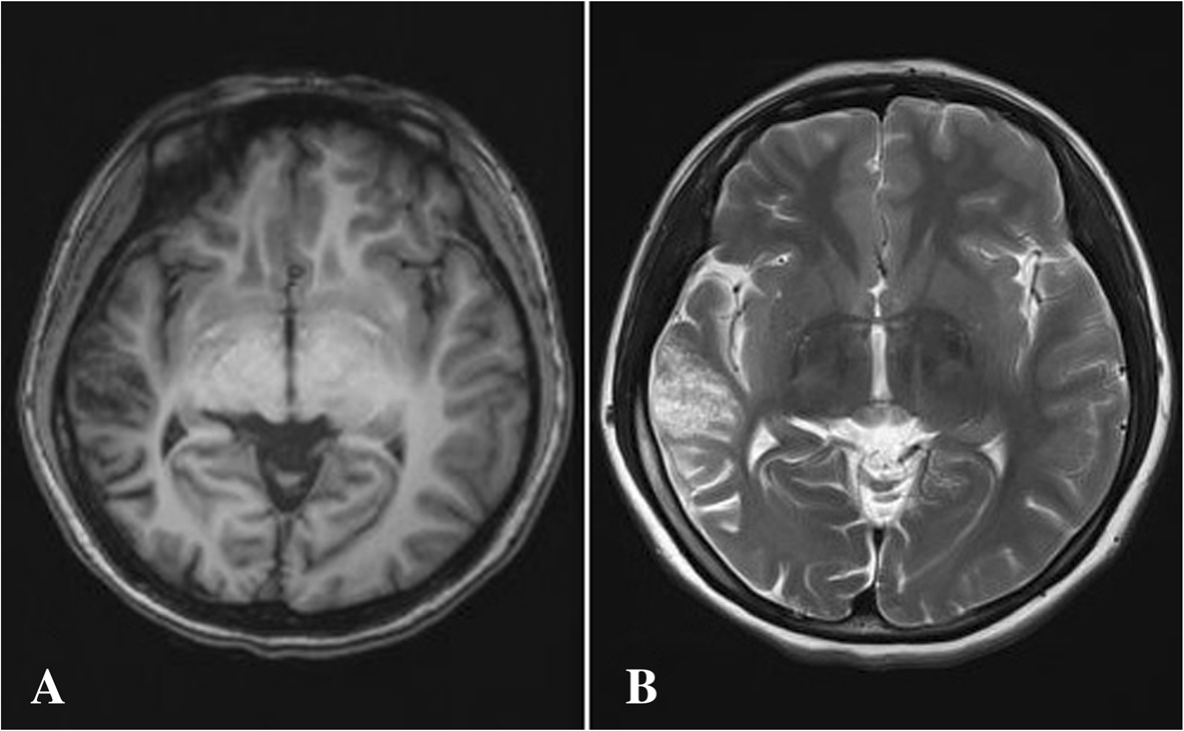
**Axial brain magnetic resonance imaging (MRI) findings.** A multicystic mass (arrow) with low signal intensity on T1-weighted images **(A)** and high signal intensity on T2-weighted images **(B)** is seen in the right temporal lobe. The cystic component was isointense with the cerebrospinal fluid on all sequences.

A 21-year-old woman presented with a history of intractable focal evolving to bilateral convulsive seizures since the age of 10 years and complained of an increase in the frequency of the seizures in the last 5 months. Eleven years ago, she experienced a complicated febrile seizure with left limb convulsion and unconsciousness, which continued for 6 h; her mental status fully recovered after 24 h. Thereafter, generalized tonic-clonic seizures occurred almost every month, in spite of medical treatment with carbamazepine, phenobarbital and sodium valproate. She had no history of adverse perinatal events, developmental problems or head trauma. General physical and neurological examinations were unremarkable; she had no stigmata or family history of NF. Inpatient continuous video electroencephalography (EEG) revealed right temporal seizure foci with significant sharp wave activity and spike-and-slow waves complex during drowsiness and sleep. Magnetic resonance imaging (MRI) of the brain demonstrated a multicystic mass with low signal intensity on T1-weighted images and high signal intensity on T2-weighted images in the right temporal lobe (Figure 1). The cysts were isointense with the cerebrospinal fluid (CSF) on T1- and T2-weighted MRI. On fluid-attenuated inversion-recovery sequence, the lesion appeared as heterogeneous hypointense mass. The lesion was well-demarcated without any perifocal edema. Clinically, a dysembryoplastic lesion was suspected. The patient underwent right temporal craniotomy and lesionectomy. Before resection, an intraoperative electrocorticography (ECoG) was performed over the lesion. The ECoG showed that epileptic foci were located in the right superior temporal gyrus, the upper part of the middle temporal gyrus and the right hippocampus. These lesions were resected under a surgical microscope. The patient was discharged 13 days after the procedure without any neurological complications. She received postoperative treatment with antiepileptic drugs for 10 months. Her postsurgical course was uneventful, and a 2-year follow-up did not reveal any recurrence of the lesion.

Gross examination of the resected right temporal lobe revealed some whitish tissue that measured 5.0 cm × 2.5 cm × 1.5 cm and had medium consistency and multiple, small, fluid-filled cysts. The right hippocampus showed some whitish tissue measuring 0.8 cm × 0.5 cm × 0.5 cm.

**Figure 2:**
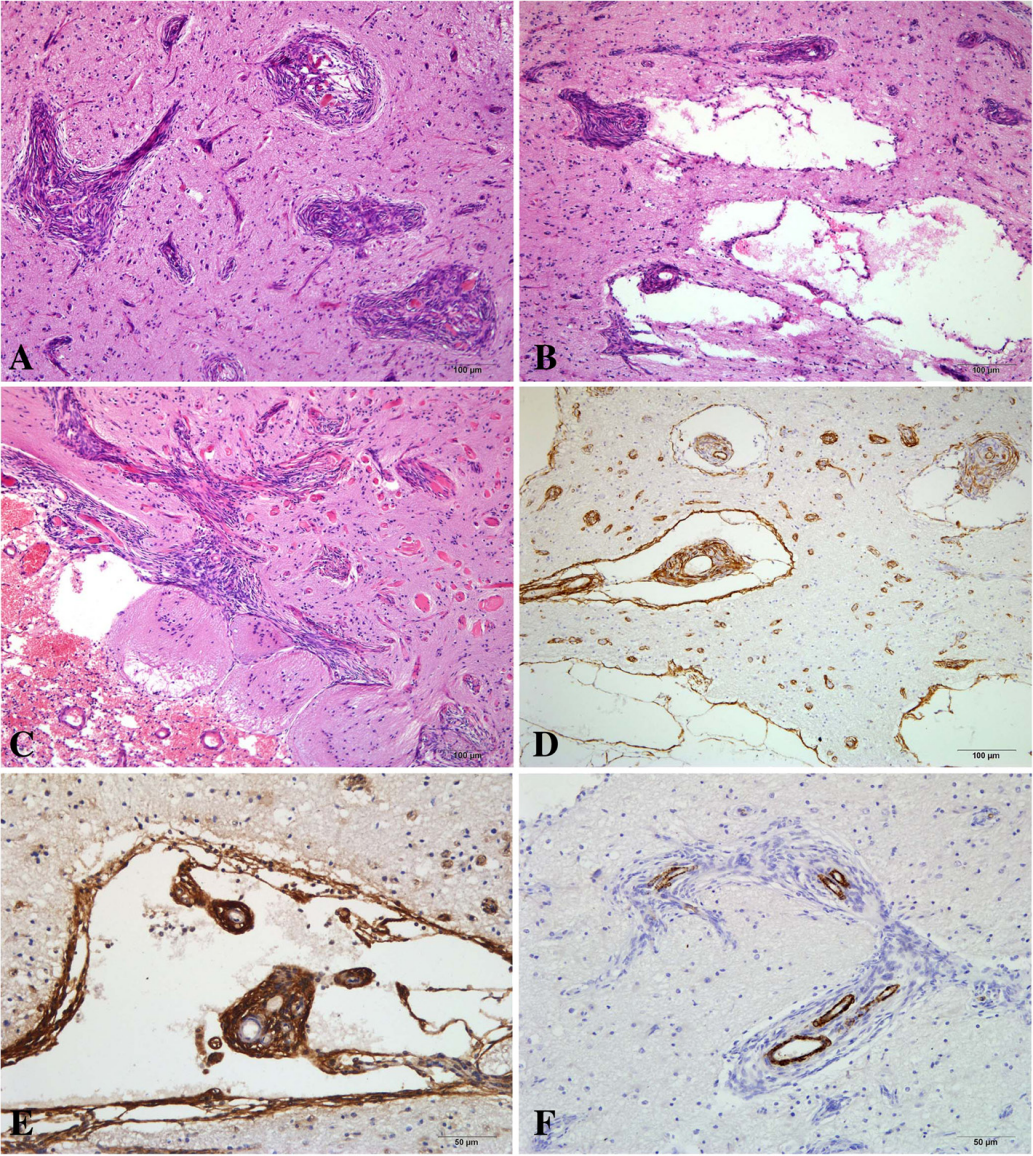
**Pathological findings of meningioangiomatosis (MA). (A)** The cortical component of the lesion shows prominent small blood vessels surrounded by perivascular meningothelial cell proliferation, which is the characteristic morphology of MA (hematoxylin-eosin, HE; ×100). **(B)** Dilated perivascular spaces and cysts are present within the perivascular cell cuffs (HE, ×100). **(C)** Cord-like nests of hyperplastic spindle cells are seen perpendicular to the surface of the cerebral cortex, where encephalocele and leptomeningeal cell proliferation are present. Hyalinized collagen fibers appear as concentrically arranged acellular eosinophilic lamellae within areas of reactive gliosis (HE, ×100). **(D)** The perivascular cells and cystic walls are focally positive for smooth muscle actin (immunohistochemistry, IHC; ×100). **(E)** The perivascular cells and cystic walls are positive for D2-40 (IHC, ×200). **(F)** The perivascular cells are negative for H-cald (IHC, ×200).

On microscopic examination, the lesion was observed to be located within the cortex and the underlying white matter, with focal involvement of the overlying leptomeninx. Pathological examination showed unique features of MA, including prominent proliferation of both the small vessels (mainly capillaries and venules) and perivascular cells, which resembled meningothelial or fibroblast cells, arranged in whorls and cuffs (Figure 2A). Microcystic components and enlarged perivascular spaces were observed within or around the perivascular cell cuffs, and some of these cysts were similar to the arachnoid cavity (Figure 2B). The perivascular cells showed direct transition into the microcystic areas or enlarged spaces, which had variable diameters and were lined with a single layer of spindle cells, but the microcysts were not connected with the vessels directly. Cord-like nests of hyperplastic fibroblast-like cells were found perpendicular to the surface of the cerebral cortex, where encephalocele and leptomeningeal cell proliferation were present. Many round eosinophilic nodules with a concentric arrangement of acellular lamellae within reactive gliosis were seen between the foci of perivascular cuffs; these nodules may have represented hyalinized collagen fibers (Figure 2C). This might have been a reactive phenomenon rather than an intrinsic MA component. Mitotic activity, cellular atypia and necrosis were not identified. In the right hippocampus, neuronal degeneration and gliosis were observed.

To determine the origin of the proliferative or lesional cells, immunohistochemical examination was performed using the Ventana BenchMark XT system (Roche Ltd., Ohio, USA). Antibodies against the following antigens were used: vimentin (clone V9; Maixin, Fuzhou, China), smooth muscle actin (SMA, clone 1A4; Dako, Carpinteria, CA, USA), epithelial membrane antigen (EMA, clone E29; Maixin), cytokeratin (CK, clone AE1/AE3; Maixin), D2-40 (clone D2-40, Dako), factor VIII (Maixin), CD31 (clone JC70A, Dako), CD34 (clone QBEnd/10; Zhongshan, Peking, China), H-cald (clone H-cald, Dako), glial fibrillary acidic protein (GFAP, clone GA-5; Maixin), neurofilament (clone 2F11, Dako) and Ki-67 protein (clone MIB-1, Dako). The perivascular cells were mainly immunopositive for vimentin and D2-40, focally or weakly positive for SMA, EMA and CD34, and negative for CK, CD31, H-cald, GFAP and neurofilament (Figure 2D,E and F). The cyst walls were often immunoreactive for vimentin, D2-40 and SMA, and less frequently for CK, H-cald and CD34. Immunoreactions with CD34, CD31 and factor VIII were detected within the cytoplasm and/or the plasmalemma of the vessels in the center of the perivascular cuffs. Ki-67 staining revealed no immunoreactive nuclei in the perivascular cells. The cerebral cortex between the perivascular cell nests was immunopositive for GFAP, neurofilament and EMA. The final diagnosis was multicystic MA.

### Discussion

MA is a rare and benign meningovascular hamartomatous condition that usually involves the cerebral cortex in the frontal or temporal regions, with varying involvement of the overlying leptomeninges. The lesions are often associated with NF2, although the majority of cases are sporadic [8]. Sporadic MA usually occurs as a solitary lesion in young patients and presents with a history of intractable seizures or persistent headaches [9,10]. In contrast, NF2-associated MA more frequently involves multiple lesions and is asymptomatic [11]. In some patients, MA may be accompanied by tumors such as meningiomas, astrocytomas and oligodendroglioma [12-14].

All MA lesions share common pathological features, namely, cortical vascular proliferation and perivascular cuffs of proliferative, spindle-shaped, meningothelial-like cells. Although approximately 120 cases of MA have been reported to date worldwide, only six cases of cysts within the MA lesion have been described in the literature (Table 1) [3-7]. The mean age of these seven patients with cystic MA including our patient was 32 years (range, 12–58 years). Except for one patient for whom insufficient data were available, all patients had intractable seizures or headaches for 2–11 years (mean, 6 years). In two patients, the cysts were visible only on microscopic examination (microcysts); in three patients, the cysts were sufficiently large to be detected on MRI. The diagnosis of MA was confirmed by the pathologist in all seven patients. Wiebe *et al*. described the histopathological spectrum of MA, which can be broadly classified into predominantly cellular and predominantly vascular types [8]. In our case, the MRI finding of a multicystic structure differentiated the condition from ordinary MA. According to the literature [1,3,6], patients with cystic MA are older than patients with non-cystic MA, and the interval between symptom onset and surgery is longer in the former. We therefore consider that cystic/microcystic MA should be classified as a new type of MA in order to better our understanding of this disease and achieve an accurate preoperative diagnosis.

**Table 1:** Summary of seven cases of cystic meningioangiomatosis

**Authors**	**Age (yrs)/sex**	**Symptoms and signs**	**Radiological findings**	**Cyst characteristics**	**Operation**	**Postoperative outcome**
Fedi M [3]	18/F	Rapidly progressing right hemiparesis and urinary incontinence	Three lesions in the left frontal cortex and subcortical white matter, a large meningioma in the foramen magnum, bilateral acoustic and right trigeminal neuromas	Small cysts within the lesions	Resection of meningioma	Died of bilateral brainstem infarction 8 months later
Wang Y [4]	12/M	Intractable seizures for 7 years	Cystic lesion in the left frontal lobe	Large cyst within the lesion	Lesionectomy	Seizures controlled with antiepileptic drugs for 10 months
Kobayashi H [5]	14/M	Intractable seizures for 3 years	A 2–3 cm mass containing small cysts in the left frontal lobe; the mass was hypointense on T1 images and isointense on T2 images	Small cysts in the periphery of the lesion	Total removal	Seizures controlled with two antiepileptic drugs for 10 months
Kuchelmeister K [6]	58/M	Headache, forgetfulness for 10 years	Multiple cysts and a meningioma in the right frontal lobe	Septated large multiple cysts	Total removal	Forgetfulness persisted; no recurrence of the lesion for 2 years after the surgery
Park MS [7]	47/F	Headache, generalized seizures for 5 years	Round calcified masses and eccentric cysts with edema in the left frontoparietal and right parietal lobes	Eccentric cysts in the left lesion	Total removal of the left lesion	No seizures or headaches for 15 months after the surgery
	53/M	Headache, generalized seizures for 2 years	Dense round calcified cysts in the left frontal and parietal lobes	Multiple macrocysts within the lesions	Total removal of both frontal and parietal lesions	No seizures or headaches for 7 months after the surgery
Author’s case	21/F	Generalized complex seizures for 11 years	Lesion showing heterogeneous signals in the right temporal lobe	Multiple microcysts within the lesion	Total surgical removal	No seizures for 2 years after the surgery

The mechanisms involved in the development of MA have not yet been elucidated. MA was considered a hamartomatous proliferation of meningothelial cells, blood vessels and fibroblasts in variable proportions [4]. However, the observation of abnormal local vascularization in some patients has led several authors to regard MA simply as a vascular malformation with an added meningothelial reaction [15]. Furthermore, it has also been suggested that these tumors do not grow or display malignant features. Some studies have supported the opinion that MA may be related to NF2 gene mutation because of its concomitance with NF2 [16]. In our patient, immunohistochemical examination demonstrated that the perivascular cells had originated from arachnoid cap cells. Considering the histopathological findings, the patient’s age at onset and the location of the lesion, we propose that MA is a hamartoma that results from the trapping of arachnoid and vascular tissue in the cerebral parenchyma during brain development, rather than a true neoplasm. The wide spectrum of immunohistochemical staining patterns observed in the perivascular cells in MA lesions in the cases reported in the literature and in our case suggests that MA originates from pluripotent cells. The exact mechanism underlying cyst development in MA remains controversial. It has been speculated that cysts form due to the accumulation of CSF within the lesion in a manner similar to the mechanism of cyst formation in cystic meningiomas [7]; however, a communication between the cysts and the subarachnoid space has not been confirmed in MA [5]. In our patient, the proliferation of meningothelial-like cells and loose perivascular cells observed on the cortical surface may have been the early stages of the typical cystic lesion in the deep cortex and white matter, and the cystic component was attributable to the enlarged perivascular spaces. The long duration of the illness supports this hypothesis. The fact that the cysts were not connected with the vessels and that the cyst contents were similar to those of the arachnoid cavity demonstrated that the cysts had originated from arachnoid tissue. Furthermore, the same immunoresponse of the perivascular cells, microcystic walls and the arachnoid cavity wall suggests that pluripotent cells differentiate into the various cell types found in MA [17] and that the formation of cysts is an accompanying developmental anomaly. Mechanisms such as increased CSF pulsation, vascular ectasia, small blood vessel obstruction or abnormal arterial wall permeability may be related to the formation of enlarged perivascular spaces, in which CSF gradually accumulates, eventually resulting in the formation of microcysts and cysts. This suggests that MA originates from pluripotent arachnoidal cap cells trapped in the cerebral cortex.

Although many cases of MA have been described, presurgical diagnosis remains difficult because the wide spectrum of clinical, imaging and electrophysiological features often impedes the clinical diagnosis [13,18,19]. Surgical resection is important not only for seizure control but also for pathological diagnosis. Wiebe *et al*. reported that after surgical removal of the tumor, long-term seizures disappeared in 43% of patients, improvement occurred in 30% of patients and antiepileptic drug administration was required in more than 70% of patients [8]. Partial removal of the tumor has been shown to improve symptoms, but total removal still seems to be more effective [8,20]. In patients in whom postoperative seizure control was not successful, extension of the epileptic foci was found to occur with time [21]. In our patient, ECoG revealed perilesional cortical spikes, and epileptogenic foci were present in not only the right temporal lobe but also the right hippocampus. Therefore, intraoperative or extraoperative ECoG is essential for detecting epileptogenic foci [22], which often exist in the adjacent cortex rather than in the lesion. In the present case, total surgical removal was achieved using intraoperative ECoG assistance, and a favorable outcome was obtained.

## Conclusion

The present case demonstrates that MA may present as a hamartomatous lesion containing multiple microcysts in a patient with medically refractory seizures, and should be considered in the differential diagnosis of intracortical lesions, especially in young adults. It is possible that MA originates from arachnoid and vascular tissue trapped in the cortical parenchyma during brain development, and that the cysts result from gradual accumulation of CSF in the perivascular spaces that develop from the trapped tissue. Our data do not support a meningeal origin of MA, but rather suggest that pluripotent arachnoid cap cells differentiate into the various cell types found in MA. Resection of the lesion and of the epileptogenic cortex is important not only for pathological diagnosis but also for seizure control, and intraoperative ECoG assistance is recommended.

## Consent

Written informed consent was obtained from the patient for publication of this case report and any accompanying images. A copy of the written consent is available for review by the Editor of this journal.

## Abbreviations

MA: Meningioangiomatosis; ECoG: Electrocorticography; NF: Neurofibromatosis; EEG: Electroencephalography; MRI: Magnetic resonance imaging; CSF: Cerebrospinal fluid; SMA: Smooth muscle actin; EMA: Epithelial membrane antigen; GFAP: Glial fibrillary acidic protein; HE: Hematoxylin-eosin.

## Competing interests

The authors declare that they have no competing of interests.

## Authors’ contributions

Acquisition of data: PL, MG and ZW. Analysis and interpretation of data: YW and GC. Drafting of the manuscript: PL, GC and YW. Critical revision of the manuscript for important intellectual content: MG and ZW. All authors read and approved the final manuscript.

## Pre-publication history

The pre-publication history for this paper can be accessed here:

http://www.biomedcentral.com/1471-2377/14/32/prepub
